# Patent Foramen Ovale in Patients with Sickle Cell Disease and Stroke: Case Presentations and Review of the Literature

**DOI:** 10.1155/2013/516705

**Published:** 2013-07-16

**Authors:** Sheila Razdan, John J. Strouse, Rakhi Naik, Sophie Lanzkron, Victor Urrutia, Jon R. Resar, Linda M. S. Resar

**Affiliations:** ^1^Hematology Division, The Johns Hopkins University School of Medicine, Baltimore, MD 21205, USA; ^2^Department of Medicine, The Johns Hopkins University School of Medicine, Baltimore, MD 21205, USA; ^3^Pediatrics, The Johns Hopkins University School of Medicine, Baltimore, MD 21205, USA; ^4^Neurology, The Johns Hopkins University School of Medicine, Baltimore, MD 21205, USA; ^5^Cardiology, The Johns Hopkins University School of Medicine, Baltimore, MD 21205, USA; ^6^Oncology, The Johns Hopkins University School of Medicine, Baltimore, MD 21205, USA; ^7^Institute for Cellular Engineering, The Johns Hopkins University School of Medicine, Baltimore, MD 21205, USA

## Abstract

Although individuals with sickle cell disease (SCD) are at increased risk for stroke, the underlying pathophysiology is incompletely understood. Intracardiac shunting via a patent foramen ovale (PFO) is associated with cryptogenic stroke in individuals without SCD. Recent evidence suggests that PFOs are associated with stroke in children with SCD, although the role of PFOs in adults with stroke and SCD is unknown. Here, we report 2 young adults with SCD, stroke, and PFOs. The first patient had hemoglobin SC and presented with a transient ischemic attack and a subsequent ischemic stroke. There was no evidence of cerebral vascular disease on imaging studies and the PFO was closed. The second patient had hemoglobin SS and two acute ischemic strokes. She had cerebral vascular disease with moyamoya in addition to a peripheral deep venous thrombosis (DVT). Chronic transfusion therapy was recommended, and the DVT was managed with warfarin. The PFO was not closed, and the patients' neurologic symptoms were stabilized. We review the literature on PFOs and stroke in SCD. Our cases and the literature review illustrate the dire need for further research to evaluate PFO as a potential risk factor for stroke in adults with SCD.

## 1. Introduction

Cerebral infarctions associated with neurologic symptoms, ischemic stroke, are relatively common in individuals with sickle cell disease (SCD). In fact, strokes are estimated to occur in about 11% of children with homozygous SCD (or hemoglobin SS) by 19 years of age [1], and the rate of stroke has been reported to be 220 times greater in children with hemoglobin SS as compared to those without SCD [2, 3]. In children, ischemic stroke is typically associated with stenosis and occlusion involving the large cerebral arteries of the Circle of Willis [4]. Silent brain infarcts, or evidence for cerebral ischemia on magnetic resonance imaging (MRI) without overt neurologic symptoms, are estimated to occur in up to 35% of individuals with hemoglobin SS [4–6]. Risk factors for ischemic stroke in children with hemoglobin SS include previous overt or silent stroke, elevated cerebral blood flow velocity, aplastic crisis, nocturnal hypoxemia, and acute chest syndrome [7, 8]. A prospective cohort study also demonstrated that the incidence of stroke is increased in older adolescents and adults with hemoglobin SS and other sickling hemoglobinopathies [1], although the incidence, pathophysiology, natural history, and associated risk factors have been less extensively studied compared to strokes in younger patients. Studies of both children and adults have identified elevated homocysteine, prior transient ischemic attack, low steady-state hemoglobin concentration, elevated systolic blood pressure or hypertension, renal disease, atrial fibrillation, hyperlipidemia, and diabetes mellitus as risk factors for ischemic stroke [1, 9, 10].

Patent foramen ovale (PFO) with intracardiac shunting is associated with cryptogenic stroke in children and young adults [11]. In individuals with a PFO, there are transient episodes of right-to-left intracardiac shunting associated with increased right atrial pressures that occur with valsalva-like maneuvers, including coughing, forced expiration, or bowel movements. Strokes in this setting are thought to occur when a venous thromboembolism travels to the cerebral arterial system after avoiding filtration in the lungs during right-to-left shunting through the PFO, an event called a paradoxical embolism [11]. Meta-analysis shows that patients with stroke, and age less than 55 years, have an increased odds ratio of 3.1 for having a PFO compared with nonstroke controls [11]. Of note, approximately 75 million people, or one-fourth of the US population, are estimated to have a PFO [12]. Since roughly 780,000 strokes occur each year, about 70,000 patients with cryptogenic stroke have a PFO. Thus, the annual risk of first ever cryptogenic stroke and PFO is estimated as one in every thousand people [12]. However, two population-based studies of first stroke in young individuals with PFO have found a nonsignificant trend towards a low but increased risk of stroke. Larger PFO diameters are associated with an increased risk for cryptogenic stroke [11–13]. Nonetheless, treatment options for PFO remain controversial due to the risks associated with both invasive and nonsurgical procedures [11, 14]. Because strokes in adults with SCD are relatively common and associated with significant neurologic sequelae, studies are needed to determine if PFOs are important risk factors in this population. 

Here, we report 2 young adults with sickling hemoglobinopathies, PFOs, and ischemic strokes. The PFO was closed in only one patient and both patients did relatively well. These cases illustrate the need for further study of stroke and PFO in adults with SCD.

## 2. Case Presentations

### 2.1. Case 1

An 18-year-old male from Nigeria with hemoglobin SC disease awoke with right-sided numbness and weakness involving the arm and leg in addition to a right-sided visual defect. The numbness and weakness resolved within a few hours, although the visual deficit persisted, prompting presentation to a local emergency department. As a child and adolescent, he had very infrequent painful crises and no other complications associated with the underlying HbSC disease. His only medication was folic acid; he was not on medications for pain. His family history, social history, and review of systems were otherwise unremarkable. The visual deficit resolved and physical examination in the emergency room was unremarkable. A complete blood count was similar to his baseline with a hemoglobin of 13.7 g/dL, hematocrit of 38.2%, and reticulocyte count of 2.0%. A CT showed no evidence for a cerebral infarction, and he was discharged with the diagnosis of a transient ischemic attack (TIA). 

**Figure 1:**
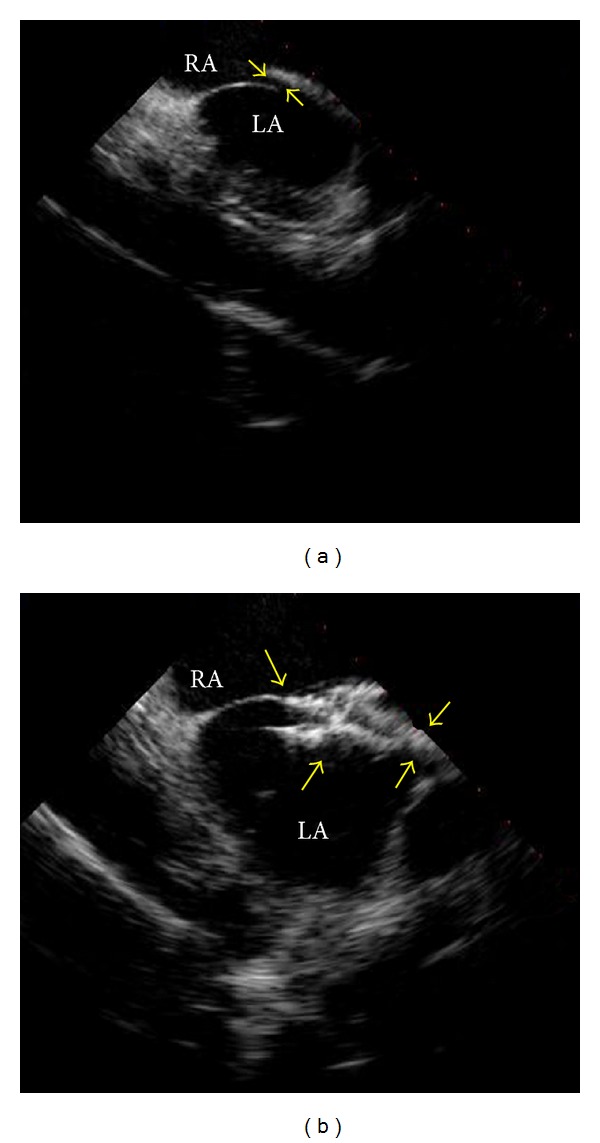
Intracardiac echocardiogram from patient 1 before and after PFO closure. (a) The intracardiac echocardiogram shows the right atrium (RA), left atrium (LA), and PFO (demarcated by arrows). (b) Intracardiac echocardiography following deployment of the closure device, also demarcated by arrows (28 mm CardioSEAL Septal Occluder; NMT Medical Inc., Boston, MA, USA).

Four months after the initial episode of neurologic symptoms, the patient developed similar visual deficits and presented to a nearby emergency department. Physical examination showed a visual field deficit of the left lower quadrant. The hematologic parameters were similar to his baseline studies; the hemoglobin was 12.7 g/dL, hematocrit was 34%, white blood cell count was 7,200, platelet count was 232,000, and reticulocyte count was 3.3%. An MRI showed a 5 mm focus of restricted diffusion in the left temporal region involving the left optic radiation, consistent with a cerebral infarct as the cause for the visual field deficit. No hemorrhage was noted, and he was admitted for further evaluation and management. He underwent an exchange transfusion to achieve a sickle hemoglobin concentration of 30%. A transthoracic echocardiogram with intravenous agitated saline demonstrated a PFO with bidirectional flow (Figure 1(a)). A doppler ultrasound of the lower extremities showed no evidence for deep venous thromboses. An evaluation for a hypercoagulable state was unremarkable with a normal antithrombin III (95%), protein C (75%), and protein S (78%) activity. There was no evidence for factor V Leiden or the prothrombin 20210 mutation. The patient was started on anticoagulation therapy with low-molecular-weight heparin and ultimately discharged on warfarin (5 mg), aspirin (325 mg), and folate (1 mg) orally each day. One month after discharge, the MRI showed no change in the size of the infarction and the MRA was normal. An evaluation for antiphospholipid antibodies (mixing studies, a dilute Russell's viper venom study, and anticardiolipin antibodies) was negative. 

Given the recurrent ischemic events in the setting of a PFO, the patient underwent percutaneous transcatheter PFO closure. A follow-up echocardiogram showed only trace shunting during vigorous coughing (Figure 1(b)). The warfarin was stopped, and he has subsequently done well without evidence of recurrent TIAs or stroke after 6 years of followup. 

### 2.2. Case 2

A 21-year-old Nigerian female with hemoglobin SS presented to a community hospital with a two-day history of left-sided headache and expressive aphasia. Her past medical history was notable for painful crises that occurred approximately once every 5 months and were managed at home with oral analgesics. Physical examination was remarkable for mild pallor and a severe expressive aphasia. She was transfused 2 units of packed red blood cells. The initial CT was normal, although an MRI showed an acute ischemic stroke in the distribution of the left middle cerebral artery. She was transferred to the Johns Hopkins Hospital for further evaluation and management. A complete blood count showed a hemoglobin of 9.9 g/dL, hematocrit of 27.4%, and reticulocyte count of 9.3%. Shortly after admission, she underwent an automated exchange transfusion to achieve a hemoglobin S concentration of 30%. The percent of hemoglobin S after the procedure was 17.0%.

A transthoracic echocardiogram and bubble study with agitated saline showed bidirectional flow consistent with a PFO. A CT angiogram showed evidence of moyamoya disease. Chronic hypertransfusion therapy was recommended, although the patient did not return for transfusion therapy. She was also placed on daily oral aspirin and folate.

Four months after the first stroke, the patient was readmitted with complaints of general malaise, weakness, intermittent confusion, and slurred speech noted four days before presentation. Physical examination was notable for a right facial droop, dysarthria, and a deviation of the tongue to the right. A complete blood count showed a hemoglobin of 11.5 g/dL, hematocrit of 32.5%, and reticulocyte count of 4.8%. The percent of hemoglobin S at presentation was 89.4%. She underwent another exchange transfusion to achieve a hemoglobin S of 30%. The day after the exchange transfusion, a doppler ultrasound of the lower extremities was performed to determine if a thrombus was present that could have provided a source for paradoxical embolus. This study demonstrated a thrombus in the common femoral vein at the site of the erythrocytapheresis catheter that had been placed the previous day for the exchange transfusion. The catheter had been removed after the exchange transfusion, and there was no leg swelling or other signs or symptoms of deep venous thrombosis (DVT). It was thought that the thrombus most likely developed in association with the catheter, and the patient was started on enoxaparin (60 mg subcutaneously every 12 hours) in addition to warfarin (5 mg orally each day). The enoxaparin was discontinued when she became therapeutic on warfarin. A hypercoagulable evaluation was done 4 days after the exchange transfusion showed a normal antithrombin III (98%) and protein C (117%). The protein S was slightly low (53%) at this time; a repeat study was recommended. There was no evidence for factor V Leiden or the prothrombin 20210 mutation. An evaluation for lupus anticoagulants and antiphospholipid antibodies (dilute Russell's viper venom test, anticardiolipin, and beta-2 glycoprotein antibodies) was negative.

This patient was managed with chronic transfusion therapy. In consultation with hematology, neurology, and cardiology, the decision was made not to repair the PFO given the small size of the PFO and the presence of significant cerebral vascular disease with moyamoya. In sickle cell disease, moyamoya is generally irreversible and often associated with ischemic strokes. She continued warfarin and was started on 40 mg of rosuvastatin for possible atherosclerotic cerebral vascular disease. She received transfusion therapy for five months with no known complications; she subsequently moved to another state and was lost to followup.

## 3. Discussion

These two patients had sickling hemoglobinopathies, PFOs, and cerebral infarcts, although there were dramatically different cerebral vascular findings on MRI/MRA. The first patient had a type of sickle cell disease (hemoglobin SC) associated with infrequent vasoocclusive crises, mild anemia, and less frequent complications. A prior study followed 145 children with hemoglobin SC for up to 18 years and did not identify any clinically overt strokes [15]. An earlier study estimated the prevalence of cerebrovascular accidents in patients with hemoglobin SC to be only 0.84% and the incidence for the first CVA to be 0.15 per 100 patient years, in contrast to the significantly greater prevalence of 4.01% and incidence of 0.61 per 100 patient years in patients with hemoglobin SS [1]. In that study, the probability of having a CVA for patients with hemoglobin SC was 2% at 20 years of age, 4% at 30 years, and 10% at 45 years [1]. The MRA in our first patient was completely normal, suggesting that there was no associated cerebral vasculopathy. It was presumed that the PFO was a major contributing factor, and he underwent a nonsurgical PFO closure via cardiac catheterization. This PFO closure approach is a relatively safe and minimally invasive procedure that is being used in patients with PFOs with or without sickle cell disease. Notably, this patient had no recurrent neurologic symptoms for 6 years despite no further transfusions or anticoagulation therapy. 

In contrast, the second patient had a more severe genotype (hemoglobin SS) and moyamoya, a severe vasculopathy whose name means “hazy puff of smoke” in Japanese [16]. Moyamoya refers to the angiographic pattern of collateral vessels that arise from the Circle of Willis after occlusions involving these vessels, which is a complication that occurs in SCD. Previous studies showed that the presence of moyamoya significantly increases the risk of recurrent stroke in pediatric patients with hemoglobin SS, even when the patients receive regular blood transfusions to maintain a low proportion of hemoglobin S [16]. After a first stroke, 43% of the cohort with moyamoya developed recurrent strokes, compared to only 28% of those without moyamoya [16]. Another study found that two out of seven patients with HbSS and neurologic deficits also presented with moyamoya [17]. Because of this significant underlying cerebral vascular disease in our second patient, chronic transfusion therapy was recommended. She also had a DVT that was managed with anticoagulation therapy. Given the potential risks for bleeding while on anticoagulation therapy and the severe cerebral vascular disease as a likely cause for the stroke, the PFO was not closed and chronic transfusion therapy was recommended. 

SCD is associated with pathologic changes and physiologic adaptations that can lead to cerebral infarction in the setting of a PFO. For example, erythrocyte adhesion, endothelial damage, and platelet and coagulation activation in SCD result in a hypercoagulable state and susceptibility to thrombosis. *In situ *microvascular thrombosis has been linked to complications such as painful crises and pulmonary hypertension [18], and there is growing evidence that the hypercoagulable state of SCD also increases the risk of venous thromboembolism. A large database study demonstrated an increased prevalence of pulmonary embolism among SCD patients compared to African-American controls [19], and the risk of DVT appears to be higher among pregnant SCD patients [20]. In addition, the prevalence of venous thromboembolism among a large adult cohort of SCD patients was 25% [21]. In the presence of PFO, the underlying hypercoagulability could lead to embolic stroke. In addition, the increased incidence of elevated pulmonary pressures in patients with SCD, either at baseline or during an episode of acute chest syndrome, could also promote right-to-left intracardiac shunting and thromboembolism to the brain in the setting of a PFO or atrial septal defect [22]. Indeed, a study in pediatric patients reported that 25% of children with hemoglobin SS and stroke also had PFOs, as compared to 11.7% of children without hemoglobin SS and stroke [23] (Table 1). These investigators concluded that further studies are warranted to determine if PFO is an independent risk factor for stroke in children with hemoglobin SS [23]. Another study in pediatric patients found that PFOs may be underestimated as possible causes of ischemic cerebrovascular events in children without sickling hemoglobinopathies [24].

Previous studies with improved imaging technology indicate that the prevalence of silent strokes for patients with sickling hemoglobinopathies is higher than reported [4]. Patients with hemoglobin SS have an 11% chance of a cerebral vascular accident by age 20, 15% by age 30, and 24% by age 45 [1]. Chronic blood transfusion therapy is typically recommended to prevent recurrent strokes in patients with sickling hemoglobinopathies and a CVA. A recent prospective study, however, found that recurrent overt or silent cerebral infarcts occur in 45% of children with hemoglobin SS and a prior ischemic stroke with a median followup of 5 years even with adequate chronic transfusions (trough HbS <30%) [25]. Importantly, both the absolute numbers and risk of death from stroke are much higher in adults than children. Indeed, 91% of the deaths resulting from stroke occur in adults [10]. These findings underscore the urgent need to assess the risk factors and potential interventions for patients with sickling hemoglobinopathies and stroke.

Future studies are needed to investigate the benefit and outcomes of nonsurgical PFO closure in patients with SCD and stroke. It will also be important to examine the relationship between SCD and PFO in both children and adults. Unfortunately, current approaches to stroke prevention for adults with SCD have been limited to secondary prevention because of the lack of methods for identifying patients at increased risk for developing their first stroke. Stroke prevention is especially important in young adults given the impact on intellectual and physical function. For young adults with SCD, stroke can lead to disability as well as reduced success in academics and employment [26], and studies have shown that children with SCD and stroke have significantly lower standardized test scores [27] and twice the rate of difficulty in school [28] compared to children with SCD without stroke. Recurrent silent infarcts, despite transfusion therapy, also lead to further cognitive deficits [25]. The devastating outcomes of acute stroke in patients with SCD demand further study, not only to define additional risk factors, but also to identify successful interventions [29–32]. Although further research is needed, the positive outcome of our patient with hemoglobin SC suggests that identifying PFOs and closing them nonsurgically could be beneficial in selected patients.

## Figures and Tables

**Table 1:** Summary of past reported studies with SCD patients with PFO and CVA.

Studies	Dowling et al., 2009 [22]	Dowling et al., 2010 [23]
[Number of patients with SCD + CVA]	1	40 (overt stroke in 30, silent infarction in 10)
Age of patients (years)	11	2–19
Number of patients with PFO	1	10
Hemoglobinopathy	HbSS	39 HbSS, 1 Hb*β* ^0^/S
Incidence of PFO	100%	25%
[Number of non-SCD patients with CVA]	N/A	60
Age of patients (years)	N/A	0.25–19
Number of patients with PFO	N/A	7
Incidence of PFO	N/A	11.7%

SCD: Sickle cell disease; PFO: patent foramen ovale; CVA: cerebrovascular accident.
